# Combination Therapy of TRAIL and Thymoquinone Induce Breast Cancer Cell Cytotoxicity-Mediated Apoptosis and Cell Cycle Arrest

**DOI:** 10.31557/APJCP.2021.22.5.1513

**Published:** 2021-05

**Authors:** Nagwa M Abdel Salam, Ahmed A. Abd-Rabou, Hayat M. Sharada, Gehan G Abd EL Samea, Mohga S. Abdalla

**Affiliations:** 1 *Egyptian Company for Blood Transfusion Services (EgyBlood), 51 Wezaret El-Zeraa Street, VACSERA, Agouza, Giza 22311, Egypt. *; 2 *Department of Hormones, Medical Research Division, National Research Centre, Cairo, Egypt. *; 3 *Department of Chemistry, Faculty of Science, Helwan University, Egypt. *

**Keywords:** TRAIL, Thymoquinone, chemo-sensitivity, breast cancer cells

## Abstract

**Objective:**

Cancer is one of the leading causes of mortality in both developed and developing nations. The tumor necrosis factor-related apoptosis-inducing ligand (TRAIL) is characterized by its ability to selectively trigger apoptosis in cancer cells. TRAIL-based interventions have led to the development of recombinant human (rhTRAIL) as a promising therapy for different types of human cancer. Thymoquinone (TQ) has been shown to exert anticancer effect. The aim of the current study is to investigate the anticancer effect of the combinatorial therapy of TRAIL+TQ against human breast cancer cells.

**Methods:**

To achieve this hypothesis, cytotoxicity using MTT assay, as well as apoptosis and cell cycle using flow cytometric technique were preceded against breast cancer MCF-7 and MDA-MB-231 cancerous cell lines.

**Results:**

The current study showed that TRAIL induced cell cycle arrest and apoptosis. Moreover, it inhibited proliferation of MDA-MB-231 cells more than MCF-7 cells. Adding TQ to TRAIL increased the chemo-sensitivity of MDA-MB-231, while overcame the MCF-7 resistance to TRAIL.

**Conclusion:**

In conclusion, there is a synergistic effect between TRAIL and TQ playing a therapeutic role in killing resistant breast cancer cells.

## Introduction

Cancer is one of the leading causes of mortality in both developed and developing nations. However, the burden of this lethal disorder is more well-known in some countries, where more than 80 % of the world population lives and the growing trend are striking (Roshandel et al., 2014). Breast cancer is known to be the second most common cause of mortality globally (Siegel et al., 2016). 

The most common treatment kinds of cancer comprise chemo-therapies. Chemotherapy drugs mainly work through injurious cellular nucleic acid. However, these types of cancer treatment contain many side effects that have a psychological, physical, and economic impact on the patient’s life (Puliyel et al., 2015). Several experimental trials have substantiated the safety, and therapeutic efficacy of the tumor necrosis factor-related apoptosis-inducing ligand (TRAIL) or TRAIL agonists in patients. TRAIL was first discovered in the 90th (Wiley et al., 1995). TRAIL is characterized by its ability to selectively trigger apoptosis in cancer cells but leaving normal cells healthy or with minimal effect (Turner et al., 2013). Qualifying as a potential drug specific for different types of cancer, containing breast, and liver (Szliszka et al., 2012; He et al., 2013; Cai Y et al., 2012; Bernardi et al., 2012); owing to its binding ability to the death receptors DR4 and DR5. Thus, TRAIL-based interventions have led to the development of recombinant human TRAIL (rhTRAIL) as a promising therapy for different types of human cancer (Strebel et al., 2001). 

The therapeutic potential of TRAIL is attributed to its receptor expression in a variety of tissues like breast, ovary, prostate, colon, and placenta compared to the restricted and transient expression of other ligands of the TNF family (Cai et al., 2012). Therefore, TRAIL is considered as a promising and effective anticancer agent under clinical investigation (Bernardi et al., 2012; Zauli et al., 2013).

Resistance to chemotherapeutic drugs is the major hindrance in the successful TRAIL cancer therapy. However, TRAIL therapy has a major limitation as a large number of the cancer develop resistance toward TRAIL and escape from the destruction by the immune system. Therefore, Combination drugs with synergistic activity are attractive in cancer therapy (Trivedi1 and Mishra, 2015). Using TRAIL in combination with Thymoquinone (TQ) as chemotherapy offers an exciting therapeutic approach that has the possibility to be rapidly translated into clinical trials.

Thymoquinone (TQ), which is one of the main constituents in Nigella Sativa, has been shown to exert anticancer effect (Woo et al., 2012). The molecular pathways of TQ as anti-cancer agent include anti-proliferation, apoptosis, and induction of cell cycle arrest and anti-angiogenesis (Majdalawieh and Fayyad, 2016; ElKhoely et al., 2015). Previous studies showed that TQ induced apoptosis and inhibited proliferation in pancreatic ductal adenocarcinoma cells (Chehlet al., 2009). Many other studies reported that TQ exhibited inhibitory effects on cell proliferation of many cancer cell lines (Chehlet al., 2012).

The development of multi-drug resistant human tumor cells including doxorubicin-resistant breast cancer cells, provoked further research with TQ to evaluate its effectiveness against this type of cells (Worthen et al., 1998; Arafa et al., 2011). They examined the anticancer effects of TQ in doxorubicin-resistant human breast cancer cells (MCF-7/DOX cells). The authors investigated the potential mechanism by which TQ may regulate cell proliferation and apoptosis in MCF-7/DOX cells. The suggested mechanism is that TQ induces apoptosis in doxorubicin-resistant breast cancer cells.

Herein, we investigate the effect of TQ and TRAIL either separately or in combination as anticancer agents on both of MDA-MB-231 and MCF-7 cells.

## Materials and Methods

*Materials*

Thymoquinone was purchased from Santa Cruz Biotechnology. TRAIL was cordially gifted from Dr. O. Micheau, INSERM, France. 3-(4, 5-dimethylthiazol-2-yl)-2,5-diphenyltetrazolium bromide (MTT) assay kit for cytotoxicity were purchased from Sigma-Aldrich (St. Louis, MO, USA). Dulbecco’s Modified Eagle Medium (DMEM), Roswell Park Memorial Institute (RPMI) 1640 medium, fetal bovine serum (FBS), penicillin/streptomycin (P/S), l-glutamine, trypsin/EDTA, phosphate-buffered saline (PBS), and annexin V/propidium iodide (PI) kit for apoptosis analysis, propidium iodide flow cytometry kit for cell cycle analysis were obtained from Life Technologies, Gibco (Grand Island, NY, USA).

*Cell Lines*

Human breast cancer MCF7 (Cat. No. HTB-22™) and MDA-MB-231 (Cat. No. HTB-26™) cell lines were purchased from cell culture department, The Egyptian Holding Company for Biological products and vaccines (VACSERA), Giza, Egypt which purchased from American Type Culture Collection (ATCC).

*In-vitro studies*

*Cell culture and maintenance*

MDA-MB-231 breast cancerous cell line is listed among the estrogen (ER), progesterone (PR), and HER2 triple-negative metastatic breast cancer panel (ATCC^® ^No. TCP-1002™). Despite both MDA-MB-231 and MCF7 cell lines are breast cancer cell lines, MDA-MB-231 is “basal” type and triple negative and MCF7 is “luminal” type and ER and PR positive cell lines. Thus, these differences affect the drug sensitivity. Breast cells were cultured using Dulbecco’s modified Eagle’s medium (DMEM) and Roswell Park Memorial Institute (RPMI-1640) medium. All media were supplemented with 4.5g/L Glucose with L-Glutamine and 10% fetal bovine serum (FBS). The cells were incubated in 5% CO_2_ humidified at 37°C for growth maintenance.

*Measurement of cytotoxicity*

All drug groups were evaluated by MTT assay (van Meerloo et al., 2011) using MCF7 and MDA-MB-231 cells. Briefly, the cells were cultured in 96-well plates at a density of 5×10^3^ cells/well. All drugs with their described concentrations were added in the media over these cell lines. Culture media with drugs (TRAIL and Thymoquinone (TQ) and without were added as control. After a day incubation, MTT dissolved in PBS was added to each well at a final concentration of 5 mg/ml, and the samples were incubated at 37°C for 4 h. Water-insoluble dark blue formazan crystals that formed during MTT cleavage in actively metabolizing cells were then dissolved in dimethyl sulfoxide (DMSO). Absorbance was measured at 455 nm, using a microplate reader (BMG Labtech, Germany). The cell viability (%) was calculated and compared with the controls. Drugs with different concentrations (0, 20, 40, 60, 80, 100 µM) for Thymoquinone (TQ) and (0, 20, 40, 60, 80, 100 nM) for TRAIL were applied upon the two cancerous cell lines for testing their toxicity levels by the following formulas:

Cytotoxicity% = 1-(Meane absorbance of toxicant)/(Meane absorbance of negative control )× 100

Viability % = 100 - Cytotoxicity %

*Apoptosis assay*

Apoptosis analysis was performed after fluorescence labeling of the cellular membrane with annexin V stain (indicating early apoptosis) and the cellular DNA with propidium iodide (PI) stain (indicating late apoptosis or necrosis). The apoptotic analysis was dedicated to differentiate between early and late apoptotic cells. Finally, the apoptosis of the MCF7 and MDA-MB-231 cells was analyzed with FACS using a flow cytometer instrument (BD Biosciences, San Jose, CA, USA).

*Cell cycle analysis*

The effect of TRAIL and Thymoquinone (TQ) on cell proliferation was evaluated by measuring the distribution of the cells in the three phases of the MCF7/ MDA-MB-231 cell cycle (G0-G1 phase, S phase, G2/M phase) by flow cytometry, using propidium iodide flow cytometry kit for cell cycle analysis. This determination was based on the measurement of the DNA content of nuclei labeled with propidium iodide (Vindelov and Christensen, 1990). Cell suspensions from either control cultures or treated cultures were prepared by trypsinization and washed with PBS. Cells (1x 10^5^ cells) were pelleted at 500Xg for 5 min, then the supernatant was discarded. Cells washed again with PBS, spined again at 500Xg for 5 min, then fixed with ethanol on ice. The cancerous cells were suspended, then pelleted at 500Xg for 5 min. The supernatant was carefully aspirated without disrupting the pellet. Cells were washed with PBS, then pellted again as before and supernatant was removed. The cells were gently resuspended in 200 µL propidium iodide and RNase staining solution, incubated at 37˚C in the dark for 30 min, then placed on ice (still in the dark). Samples were run on BD flow cytometer. 

*Statistical analysis*

All assays were repeated three times. Comparisons between different groups versus controls were made using a two-tailed Student’s t test, and values of P < 0.05 were considered statistically significant.

## Results

*Cytotoxicity of combination therapy of TRAIL and TQ*

The present study was designed to evaluate the chemotherapeutic activity of TRAIL and Thymoquinone (TQ) as a combinatorial anticancer agent and define the most effective approach of application. Determination of anticancer potential of TRAIL and/or TQ against human breast cancer cell lines (MDA-MB-231 and MCF-7) by performing cytotoxicity assay to detect the reduction of MTT by mitochondrial dehydrogenase, which reflects the normal function of mitochondria and hence the measurement of cytotoxicity and cell viability.

**Figure 1:**
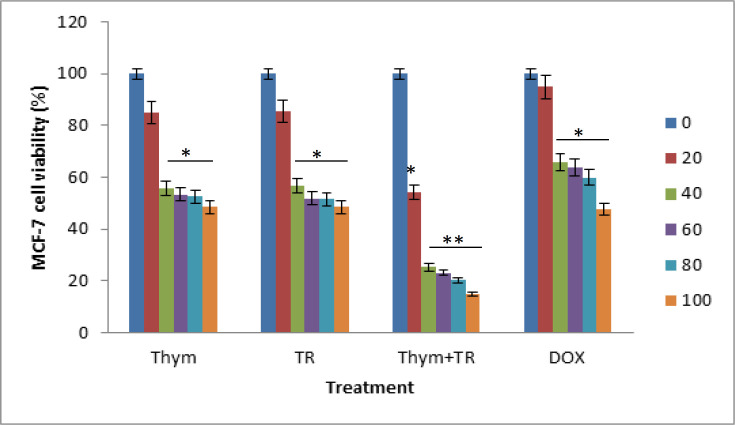
Cell Viability Determined after Treatment of MCF-7 Cell Line with Serial Concentrations of Thymoquinone (Thym= TQ) and TRAIL (TR). TQ concentrations were (0, 20,40, 60, 80 and 100 μM) and TR concentrations were (0, 20,40, 60, 80 and 100 nM) and their combination using MTT assay. Data were measured after 24 h using Doxorubicin (DOX) as a positive control with serial concentrations (0, 20,40, 60, 80 and 100 μM). * means significant difference (p less than 0.05) compared to control, ** means high significant difference (p less than 0.01) compared to control. TRAIL, tumor necrosis factor-related apoptosis inducing ligand; TQ, thymoquinone

**Figure 2:**
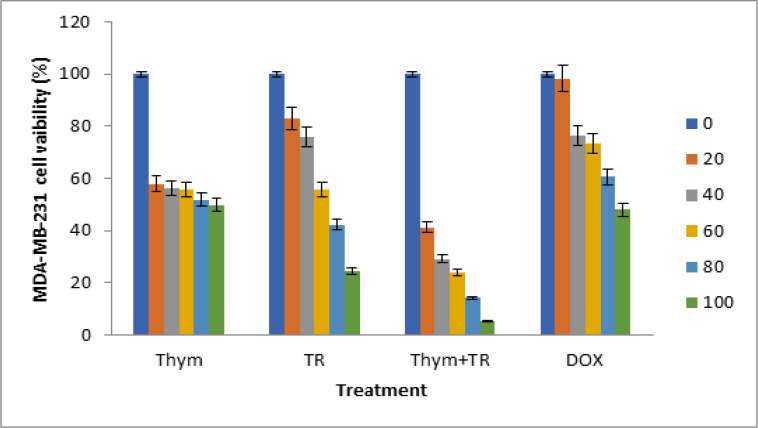
Cell Viability Determined after Treatment of MDA-MB-231 Cells with Serial Concentrations of Thymoquinone (Thym= TQ) and TRAIL (TR). TQ concentrations were (0, 20,40, 60, 80 and 100 μM) and TR concentrations were (0, 20,40, 60, 80 and 100 nM) and their combination using MTT assay. Data were measured after24 h using Doxorubicin (DOX) as a positive control with serial concentrations (0, 20,40, 60, 80 and 100 μM). * means significant difference (p less than 0.05) compared to control, ** means high significant difference (p less than 0.01) compared to control

TRAIL, tumor necrosis factor-related apoptosis inducing ligand; TQ, thymoquinone.

We initially studied the effect of TQ on the breast cancer cell death-mediated apoptosis to investigate the effects of TQ on cancer cell growth, which showed that TQ can decrease the cell growth of both MDA-MB-231 and MCF-7 cancerous cells (Figures 1, 2). Using Doxorubicin (DOX) as a positive control anti-cancer drug, the doxorubicin concentrations used in the study 0, 20, 40, 60, 80, 100 μM in parallel with the proposed treatments.

There was a slight effect of TQ on MCF-7 breast cancerous cells at 20 μM dose only 17.56% reduction in MCF-7 cell viability after 24 h. We found significant reduction effects of TQ; 46.56, 47.39, 47.39, 44.08% in MCF-7 cell viability after 24 h (p < 0.05). These results clearly show the ability of TQ to alter the cancerous cellular viability.

However, in MDA-MB-231 cell line, there was a slight effect of TQ on MDA-MB-231 cells at 20 μM conc, 42.02% reduction in MDA-MB-231 cell viability after 24 h. Additionally, we found significant effects of TQ; 43.72, 44.72, 48.04, 50%, respectively reduction in MDA-MB-231 cell proliferation after 24 h (p < 0.05). 

These results clearly confirm the ability of TQ to alter the MDA-MB-231 cellular viability. These indicate that treatment of TQ shows significant cytotoxic effects on MCF-7 and MDA-MB-231 breast cancerous cell lines, suggesting that MDA-MB-231 more sensitive to thymoquinone than MCF-7 breast cancerous cells.

We then studied the effect of TRAIL on breast cancer cell in both of MDA-MB-231 and MCF-7 cells to investigate the effects of TRAIL on cancer cell growth, showing that TRAIL may reduce growth of both MDA-MB-231 and MCF-7 cancerous cells (Figures 1,2).

There was low effect of TRAIL on MCF-7 cancerous cells at 20 nM dosage, only 19.35% reduction in MCF-7 cell viability after 24 h incubation. On the other hand, we found significant effects of TRAIL 48.35, 48.08, 51.38, 43.39% respectively reduction in MCF-7 cell proliferation after 24 h (p < 0.05). However, in MDA-MB-231 there was no clear effect of TRAIL on MDA-MB-231 cells at 20 μM dosage only 5% reduction in MDA-MB-231 cell viability after 24 h. Also, we found significant effects of TRAIL 16, 28, 44.25, 43% respectively reduction in MDA-MB-231 cell proliferation after 24 h (p < 0.05). 

The cell viability assay showed that MCF-7 cells more sensitive to TRAIL than MDA-MB-231 (Figures 1,2). Studying the effect of the combination of TRAIL and thymoquinone treatment on MCF-7cells showed higher significant effects 45.74, 74.74, 79.69, 76.08, 85% respectively reduction in MCF-7 cell proliferation after 24 h compared to that of the individual treatments.

However, in MDA-MB-231 cells the effect of the combination of TRAIL and thymoquinone treatment was highly significant 58.63, 60.72, 61.9, 65.83, 81% respectively reduction in MDA-MB-231cell proliferation after 24 h compared to that of the individual treatments.

*The IC*_50_ *and fold change of combination therapy of TRAIL and TQ*

IC_50_ values and fold change of drug-resistant cell models is developed in the laboratory by repeatedly exposing MDA-MB-231 and MCF-7 cancerous cells growing in cell culture to drugs. The surviving daughter resistant cells were then compared to the parental sensitive cells using combination cell viability/proliferation assays using the MTT. The sensitivity of these paired cell lines were usually determined by exposing them to a range of TRAIL and thymoquinone concentrations and then assessing cell viability. The IC_50 _(drug concentration causing 50% growth inhibition) for these paired cell lines will be used to determine the increase in resistance known as fold resistance by the following equation: 

Fold Resistance= IC_50_ of Resistant Cell Line∕ IC_50_ of Parental Cell Line

After applying this equation, we found that the combination of TRAIL and thymoquinone reduces cell viability in both MCF-7 and MDA-MB-231, but MCF-7 more sensitive to the combination of TRAIL and thymoquinone than MDA-MB-231. In the present investigation, the combination of TRAIL and thymoquinone enhance their ability to inhibit the proliferation of breast cancer cells in-vitro (Tables 1,2). 

This mean that the combination of TRAIL and thymoquinone considered as promising drugs for treatment of breast cancer MCF-7 and MDA-MB-231 cell lines. It could be concluded that these combinations are effective at low dose rates, with controlled and effective against resistant breast cancer and could be applied as an anticancer strategy.

The cytotoxic effect of thymoquinone and TRAIL monitored and the resulted IC_50_ values were 69.94 μM and 67.72 nM respectively in case of MCF-7 treated cell, while the IC_50 _values of the combination of TRAIL and thymoquinone on the same cell line were 25.81 μM and 25.81 nM respectively (Table 1). 

Furthermore, for treatment of MDA-MB-231 cancerous cell line with thymoquinone and TRAIL, the IC_50_ values were 56.067 μM and 69.66 nM respectively, and the IC_50_ value of the combination of TRAIL and thymoquinone was 24.64 μM these mean that the combination is effective at low dose rates, to confirm that a combination is necessary for inducing profound cell death.

We determined the cell viability inhibition concentration (IC_50_ values) of TRAIL on cancer cells with or without Thymoquinone. When cells were treated with TRAIL alone, IC_50_ values could be obtained only on TRAIL-sensitive MCF-7 and MDA-MB-231cell line. After combination with Thymoquinone, the potency of TRAIL increased >1.44 -fold change(lower IC_50_ values) in MCF-7 and increased 1.39-fold change in MDA-MB-231cell line, the potency of Thymoquinone increased 1.40–fold change (lower IC_50_ values) in MCF-7 and increased 1.39-fold change in MDA-MB-231cell line, the potency the combination of Thymoquinone and TRAIL increased 3.79 –fold change (lower IC_50_ values) in MCF-7 and increased 3.94-fold change in MDA-MB-231 breast cancerous cell line (Tables 1,2).

*Coefficient of drug interaction (CDI)*

The coefficient of drug interaction was used to analyze the synergistically inhibitory effect of the drug combination. CDI was calculated as follows: CDI=AB/(A×B).

AB is the ratio of the two drugs combination group to the control group and A or B is the ratio of the single drug group to the control group.

CDI <1 indicates synergism, especially CDI <0.7 indicates a significantly synergistic effect, CDI = 1 indicates additive.

MTT assay for cell viability CDI for MCF-7 = 25.51/56.45*66.03 = 0.00674

MTT assay for cell viability CDI for MDA-MB-231= 30.36/99.98*56.03 = 0.00542

In this study, a new combination of TQ with TRAIL was assessed for treatment of MCF-7 and MDA-MB-231cell line. By calculating the CDI ratio, the ratio was less than 0.7 showing a significant synergistic effect when combining TQ and TRAIL.

*Apoptosis*

**Figure 3:**
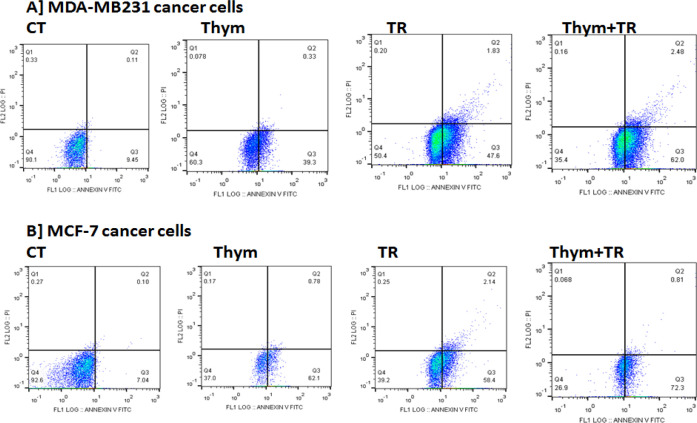
Apoptosis Using Fow Cytometry. Flow cytometric analyses of MDA-MB-231 and MCF-7 cell lines were performed for early and late apoptotic upon treatments with the IC_50_ of Thym (TQ), TR, and Thym (TQ)+TR versus control

We next determined the mechanism by which TQ and TRAIL kills MDA-MB-231 and MCF-7 breast cancerous cells. We observed an enhanced apoptosis of MDA-MB-231 cells treated with Thym (TQ)+TRAIL compared with TRAIL and Thym (TQ) (Figure 3). In MCF-7 cells, we found that the combination therapy of Thym (TQ)+TRAIL induce 72.3% early apoptotic cell death compared to 58.4% early apoptotic cell death upon TRAIL treatment and 62.1% early apoptotic cell death upon Thym (TQ) treatment, compared to the control untreated cells. In MDA-MB-231cells, we found that the combination therapy of Thym (TQ)+TRAIL induce 62.0% early apoptotic cell death compared to 47.6% early apoptotic cell death upon TRAIL treatment and 39.3% early apoptotic cell death upon Thym (TQ) treatment, compared to the control untreated cells.

*Cell cycle analysis*

**Figure 4:**
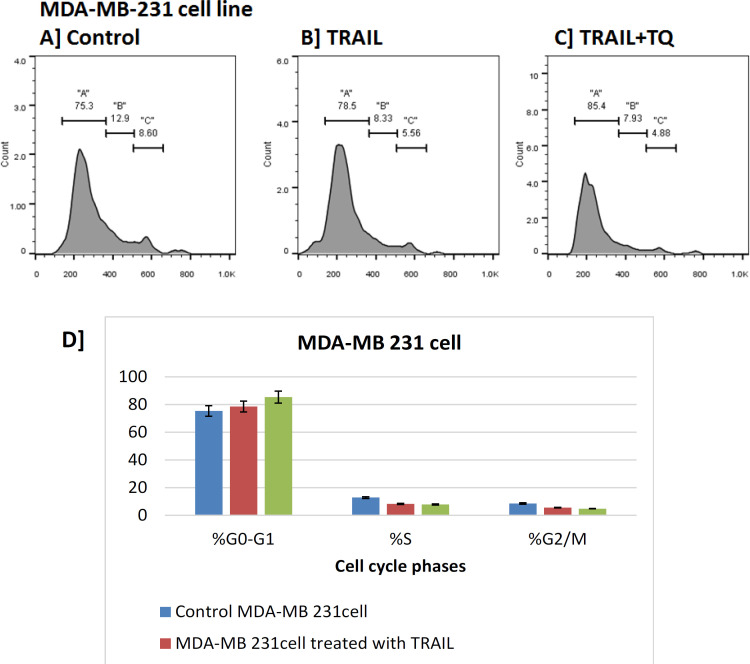
Cell Cycle Analyses of MDA-MB-231 Cells Using Flow Cytometry. Flow cytometric analyses of MDA-MB-231 cell line was performed for G0/G1, S, and G2/M phases upon treatments with the IC_50_ of TR (B) and TR+TQ (C) versus control (A). D) the results were repeated 3 times (n=3) and the graphical diagram in percentage (%) of G0/G1, S, and G2/M phases were illustrated. A, G0/G1; B, S; C, G2/M phases

**Figure 5:**
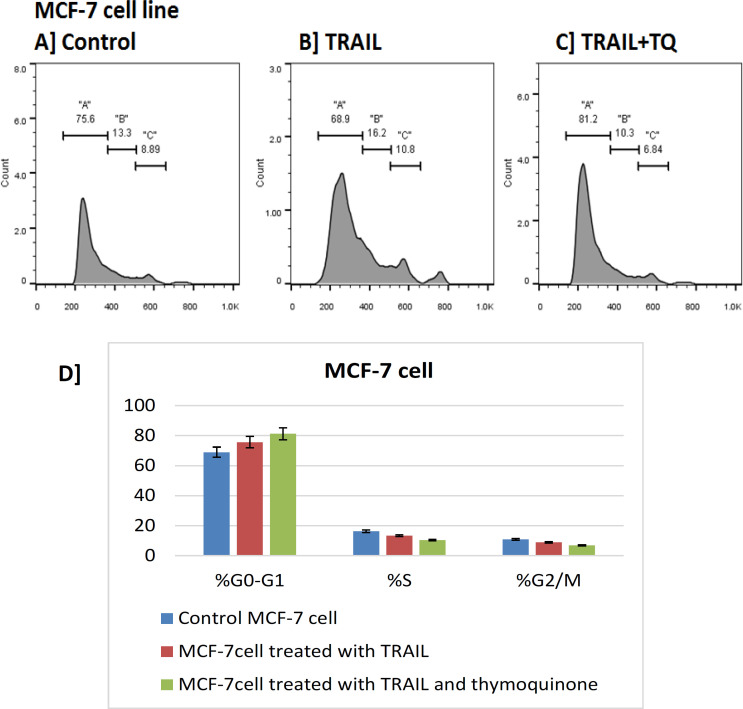
Cell Cycle Analyses of MCF-7 Cells Using Flow Cytometry. Flow cytometric analyses of MDA-MB-231 cell line was performed for G0/G1, S, and G2/M phases upon treatments with the IC_50_ of TR (B) and TR+TQ (C) versus control (A). D) the results were repeated 3 times (n=3) and the graphical diagram in percentage (%) of G0/G1, S, and G2/M phases were illustrated. A, G0/G1; B, S; C, G2/M phases

It was observed that the percentage of S% phase and G2-M% were reduced compared to the control in both MCF-7 and MDA-MB-231 breast cancerous cell lines treated with the combination therapy of thymoquinone and TRAIL, while the percentage of G0-G1% was increased (G0-G1% phase arrest) in both cells treated with both compounds, which indicate the positive effect of test compounds on these cancer cells in-vitro. The cell cycle analysis confirmed that TRAIL induced a depletion of cells in the G0-G1% phase; meanwhile, S phase cell cycle arrest (Figures 4,5).

**Table 1:** The IC_50_ and Cytotoxicity Fold Change vs DOX against MCF-7 Cell Line

	Thym	TR	Thym+TR	DOX
IC_50_	69.94	67.72	25.80	97.94
Dose:100	48.61	48.62	15.00	47.62
Cytotoxicity fold change vs DOX	1.40	1.44	3.79	Ref.

MCF-7 cell line was treated for 24 h, IC_50_ values shown in (µM) for Thym or DOX as well as in (nM) for TRAIL. The IC50 values, maximum cell proliferation at 100 µM, and Cytotoxicity fold change of Thym , TR, and Thym+TR versus DOX were shown.

**Table 2:** The IC_50_ and Cytotoxicity Fold Change vs DOX against MDA-MB-231 Cell Line

	Thym	TR	Thym+TR	DOX
IC_50_	56.06	69.66	24.64	97.11
Dose:100	49.80	24.3	5.20	48.00
Cytotoxicity fold change vs DOX	1.73	1.39	3.94	Ref.

MDA-MB-231 cell line was treated for 24 h, IC_50_ values shown in (µM) for Thym or DOX as well as in (nM) for TRAIL. The IC50 values, maximum cell proliferation at 100 µM, and Cytotoxicity fold change of Thym , TR, and Thym+TR versus DOX were shown.

## Discussion

Cancer drug resistance has been reported to develop novel combinatorial treatment. The synergistic effect of TRAIL when combined with an anticancer agent is a promising strategy to overcome the resistance of cancerous cells toward TRAIL. For instance, the combinatorial treatment of chrysin and TRAIL considerably augments apoptosis in lung cancer cells (Mehdi et al., 2019).

However, many cancerous cells display resistance to TRAIL, susceptible cancerous cells can easily acquire resistance. Thus, we proposed TQ that can synergize with TRAIL in sensitive breast cancerous cells. The TQ can alter the cell cycle progression and induce cell death independent of FASN mediated signaling (Arif et al., 2019). The TQ chemo-combination therapeutics increased the modulation to the anticancer consequence and protected the healthy cells from the cytotoxic burdens that are induced by the chemotherapy based (Ali et al., 2020). The question arises whether these agents can also work effectively with TRAIL in resistant breast cancerous cells.

Recent results of randomized Phase II trials of TRAIL R-targeted drugs reported at the 2010 ASCO meeting were not encouraging; combinations with cytotoxic chemotherapy with or without chemotherapy did not demonstrate additional benefit from the TRAIL R targeted drug in non-small cell lung cancer (NSCLC) patients (Blackhall et al., 2010; VonPawel et al., 2010; Karapetis et al., 2010).

Thymoquinone (TQ) is a promising natural compound with significant in vitro and in vivo antineoplastic activities against different tumor cell lines. It is a potent inducer of cell cycle arrest and apoptosis. To solve the problem of cancer chemo-resistance, we added the TQ to the TRAIL for sensitizing the cancerous cells. Preclinical studies reveal the potential of TQ in improving the therapeutic effect of anticancer drugs and also protection of non-tumor tissues against chemotherapy-induced damages (El-Baba et al., 2014).

TRAIL was previously reported as having hepatic protective effect and antitumor activity. In the current study, the IC_50_s were found to be 67.72 μM and 69.66 nM for MCF-7 and MDA-MB-231 breast cancerous cells, respectively. 

In this regard TQ has been assessed on MCF-7 and MDA-MB-231 cell lines. We demonstrated that TQ has significant cell viability and inhibitory effect on MCF-7 and MDA-MB-231 cell lines. Our results were in agreement with previous studies of Salim’s group who found a remarkable decrease in viability of lymphocyte leukemia cells when treated with TQ, suggesting that thymoquinone inhibited cell proliferation compared to the control untreated group (Salim et al., 2013). 

Our results are also in agreement with the studies of Ismail’ team who investigated the effect of thymoquinone alone and in combination with resveratrol on hepatocellular carcinoma HepG2 cells (Ismail et al., 2013), and Salim (2013) who found a decrease in viability of lymphocyte leukemia cells when treated with Thymoquinone (Salim et al., 2013). 

Our data showed that our combinatorial drugs achieving synergy upon MCF-7 and MDA-MB-231 cancerous cell lines. The potency of TRAIL increased 1.44 -fold change (lower IC_50_ values) in MCF-7 and increased 1.39-fold change in MDA-MB-231 cell line, the potency of Thymoquinone increased 1.40 –fold change (lower IC_50_ values) in MCF-7 and increased 1.39-fold change in MDA-MB-231cell line, the potency of the combination of Thymoquinone and TRAIL increased 3.79 -foldchange (lower IC_50_ values) in MCF-7 and increased 3.94-fold change in MDA-MB-231cell line.

Other combinations, as thymoquinone and resveratrolwere were reported as a promising combination when added together and enhanced the anticancer effect. In this study, a new combination of TQ with TRAIL was assessed for treatment of MCF-7 and MDA-MB-231cell lines. By calculating the CDI ratio, the ratio was less than 0.7 showing a significant synergistic effect when combining TQ and TRAIL. This combination strategy shows promise in cancer therapy and need further investigation to develop a better treatment strategy.

Norsharina and companions used thymoquinone rich fraction extracted from Nigella sativa and commercially available thymoquinone on colon cancer (HT29), lymphoblastic leukemia (CEMSS) and promyelocytic leukemia (HL60) cells lines. In agreement with our study, they found a cell cycle arrest and an increment of apoptosis in a time dependent manner for all cell types (Norsharina et al., 2011).

In this study, the analysis of cell cycle of MCF-7 and MDA-MB-231 cells treated with TRAIL compared with control cell showed significant increase of cell population at G0/G1 phase, and a significant decrease in cell population at S phase and mitosis (G2/M phase). The combination of TQ and TRAIL treatment showed more significant increase of cell population at G0/G1 phase, and a significant decrease in cell population at S phase and mitosis (G2/M phase). These mean cells were arrested in G0/G1 phase, which implies that thymoquinone can partially reverse the drug resistance of MCF-7 and MDA-MB-231 cells.

Our result suggested that thymoquinonein combination with TRAIL may be a therapeutic agent against MCF-7 and MDA-MB-231 breast cancer cells, which may be get attention in near future as a breakthrough in the treatment of breast cancer patients.

In conclusion, the combination of TQ and TRAIL showed a promising outcome. TQ and TRAIL enhanced cell viability inhibition occurred with compared to TQ and TRAIL individually. This study showed that TQ and TRAIL are promising cytotoxic agents and their combination upon MCF-7 and MDA-MB cells showed potent anti-cancer efficacy and pro-apoptotic effect-mediated cell cycle arrest.

## Author Contribution Statement

All authors contributed in this work. Sharada, Abd EL Samea, Abdalla, and Abd-Rabou are the supervisors of the NM Abdel Salam’s PhD thesis. Abd-Rabou had the work idea and was responsible for paper writing and editing, with Abdel Salam, and publishing it as well as the cell culture processing and applications. Abdel Salam, Sharada, Abd EL Samea, and Abdalla shared in all experiments and edited the manuscript.
